# 石墨氮化碳/金属有机骨架萃取纤维用于富集肺癌患者呼出气中挥发性有机化合物

**DOI:** 10.3724/SP.J.1123.2024.03002

**Published:** 2024-12-08

**Authors:** Qilong HAO, Jing WANG, Liqing YU, Haixia ZHANG

**Affiliations:** 1.河北大学化学与材料科学学院,河北 保定 071002; 1. College of Chemistry and Materials Science, Hebei University, Baoding 071002, China; 2.兰州大学化学化工学院,甘肃 兰州 730000; 2. College of Chemistry and Chemical Engineering, Lanzhou University, Lanzhou 730000, China

**Keywords:** 挥发性有机化合物, 固相微萃取, 气相色谱-质谱, 生物标志物, 早期诊断, volatile organic compounds (VOCs), solid-phase microextraction (SPME), gas chromatography-mass spectrometry (GC-MS), biomarkers, early diagnosis

## Abstract

人体呼出气中的挥发性有机化合物(VOCs)可作为评价生理或病理条件的生化指标。然而,呼出气成分复杂,且VOCs含量较低,难以达到分析仪器的直接检测要求。因此,建立一种简便、快捷、高效的富集技术对VOCs的直接检测具有重要意义。该文将石墨氮化碳(g-C_3_N_4_)与金属有机骨架复合,制备了三维纳米花状的g-C_3_N_4_/UiO-66固相微萃取涂层,联用GC-MS技术对肺癌患者呼出气中的5种生物标志物(苯、甲苯、乙苯、邻二甲苯和癸醛)进行富集检测。通过FT-IR、TGA、XRD和SEM对复合材料进行系列表征,纳米花状固相微萃取纤维的最佳萃取时间为10 min,最佳萃取温度为60 ℃,最佳解吸时间和解吸温度分别为3 min和260 ℃。 结果表明,5种VOCs在各自的浓度范围内具有良好的线性关系,相关系数为0.9919~0.9977,检出限为0.16~4.50 μg/L,定量限为0.54~9.18 μg/L。纤维6次平行测定的日内和日间精密度分别为3.3%~7.3%和4.0%~6.6%。由于复合材料中纳米花状层间通道以及g-C_3_N_4_微孔的存在,该复合材料涂层表现出较高的富集效率和良好的重复性。5种VOCs的加标回收率稳定在84.6%~113.2%范围内。最后,将该方法应用于肺癌患者呼出气分析检测,无论是健康人群还是肺癌患者,呼出气中苯的含量普遍较低,甲苯的含量差异性明显且肺癌患者远高于健康人群。所建立的方法有望为肺癌无创早期诊断提供参考价值。

## Abstract (en)

Volatile organic compounds (VOCs) in human exhaled breath can act as biochemical indicators for the objective determination and evaluation of physiological or pathological conditions. In humans, the pathophysiology associated with malignant tumors causes metabolic changes that alter the types and concentrations of VOCs in exhaled breath. However, as exhaled breath has a complex composition and low VOC content, meeting the direct detection requirements of analytical instruments is difficult. Therefore, the direct detection of VOCs in exhaled breath requires the development of a simple, fast, and efficient enrichment technique. In this study, graphitic carbon nitride (g-C_3_N_4_) and the metal-organic framework UiO-66 were combined to prepare a three-dimensional nanoflower-like g-C_3_N_4_/UiO-66 solid-phase microextraction (SPME) coating. The g-C_3_N_4_/UiO-66 composite was characterized using Fourier transform infrared spectroscopy, thermogravimetric analysis, X-ray diffraction, and scanning electron microscopy. UiO-66, which exhibited a regular ortho-octahedral structure with sharp corners, was embedded among g-C_3_N_4_ nanoflowers, and the surface was encapsulated in g-C_3_N_4_ to form a hydrophobic barrier. Five biomarkers (benzene, toluene, ethylbenzene, *o*-xylene, and decanal) in the exhaled breath of patients with lung cancer were enriched using the developed SPME fiber and then detected using gas chromatography-mass spectrometry. The optimum extraction time and temperature of the nanoflower-like SPME fiber were 10 min and 60 ℃, respectively, whereas the optimum desorption time and temperature were 3 min and 260 ℃, respectively. The adsorption efficiencies of the g-C_3_N_4_/UiO-66 composite coating, a single UiO-66 coating, and a 30 μm polydimethylsiloxane commercial coating were compared under optimal conditions. The five VOCs showed good linearity in the investigated concentration ranges, with correlation coefficients of 0.9919-0.9977, limits of detection of 0.16-4.50 μg/L, and limits of quantification of 0.54-9.18 μg/L. The intra- and inter-day precisions for six parallel determinations with the SPME fiber were 3.3%-7.3% and 4.0%-6.6%, respectively. Owing to the presence of nanoflower-like interlayer channels and g-C_3_N_4_ micropores in g-C_3_N_4_/UiO-66, the composite coating exhibited a high enrichment efficiency and good repeatability. The spiked recoveries of the five VOCs were in the range of 84.6%-113.2%. Finally, the method was applied to analyze exhaled breath for the detection of lung cancer. The benzene contents in the exhaled breath of both healthy individuals and patients with lung cancer were generally low. However, the toluene contents differed significantly, with patients with lung cancer having much higher levels than healthy individuals. The established method is expected to provide a reference for the noninvasive early diagnosis of lung cancer.

人体呼出气中的挥发性有机化合物(VOCs)是客观测定和评价生理或病理条件的生化指标,这些VOCs在生理或代谢过程中产生,既能直接通过呼气方式释放,又可以参与血液循环,通过肺泡肺膜释放到呼出气中^[1,2]^。科学研究发现,呼出气中的VOCs浓度差异与人体健康状况有关^[3⇓-5]^。人体在出现恶性肿瘤时,其病理生理引起代谢变化,肿瘤细胞将产生反应疾病状况的独特VOCs,从而导致VOCs的组成和浓度发生改变^[6]^。因此,通过检测不同人群呼出气中具有癌症特征的VOCs浓度值,可以实现癌症早期筛查和诊断^[7⇓-9]^。呼出气检测技术具有非侵入式、样品采集快速便捷等优点,然而,呼出气成分复杂,且VOCs含量较低(一般为μg/L或ng/L)。因此,在分析检测呼出气中的VOCs前需要一种简便、快捷、高效的富集技术。

固相微萃取(SPME)技术于1990年由Pawliszyn团队首次提出^[10]^,该技术集采样、萃取、富集、进样于一体,解决了使用有机溶剂对环境造成危害的问题,避免了多步提取的繁琐操作^[11]^,且采用SPME的样品制备非常简单,尽可能地避免了在分析过程中引入不必要的误差,从而获得更准确可靠的分析结果^[12]^。目前,SPME技术已广泛用于环境^[13]^、法医鉴定^[14]^、毒理学^[15]^、食品风味^[16]^、制药^[17]^和农药残留检测^[18,19]^等诸多领域。由于SPME技术的选择性和灵敏度主要依赖于目标物在萃取纤维上的吸附解吸过程。因此,开发新型的吸附剂涂层材料是SPME技术的研究焦点^[20]^。

金属有机骨架(MOFs)因其超高的比表面积和孔隙率、可调节的化学成分以及优异的可定制性,在各个领域都有广阔的应用前景^[21⇓⇓-24]^。但纵观研究学者对MOFs在SPME领域中的应用研究,其结果仍有不尽人意的地方,比如稳定性差,特别是湿敏感性,阻碍了MOFs的广泛应用^[25⇓-27]^。石墨氮化碳(g-C_3_N_4_)是石墨烯的类似物,具有富电子石墨结构和游离的氨基(-NH_2_、-NH-),可以通过*π-π*共轭、氢键、静电以及疏水相互作用等方式吸附缺电子化合物^[28]^。此外,g-C_3_N_4_还具有优异的机械强度和热稳定性。如果利用MOF和g-C_3_N_4_结构可调节的特性按一定比例结合形成g-C_3_N_4_/MOF复合材料,不但能够弥补材料单相应用时的不足,甚至还有“1+1>2”的协同效果。

本文通过制备一种高选择性和吸附性的g-C_3_N_4_/UiO-66复合涂层萃取纤维,对肺癌患者呼出气中的5种生物标志物(苯、甲苯、乙苯、邻二甲苯和癸醛)进行固相微萃取研究。考察了萃取时间、萃取温度、解吸时间和解吸温度对萃取效率的影响。在最佳实验条件下,用自制的g-C_3_N_4_/UiO-66复合涂层纤维对呼出气实际样品中的生物标志物进行了富集研究,并采用气相色谱-质谱(GC-MS)联用技术进行了分析测定。本研究有望为肺癌早期诊断和筛查开辟新的前景。

## 1 实验部分

### 1.1 仪器、试剂与材料

气相色谱-质谱联用仪(GC-Trace 1300-ISQ LT,美国赛默飞世尔科技公司),X射线粉末衍射仪(XRD, D8 ADVANCE,德国布鲁克科技公司),热重分析仪(TGA, STA449C/QMS403C,德国耐驰仪器制造有限公司),傅里叶变换红外光谱仪(FT-IR, Nicolet iS10,德国布鲁克科技公司),冷场扫描电子显微镜 (SEM, JSM-7500,日本电子公司)。

氯化锆、邻二甲苯(分析纯,上海麦克林生化科技有限公司),对苯二甲酸、尿素、癸醛(分析纯,上海阿拉丁化学试剂科技有限公司), *N*,*N*-二甲基甲酰胺(DMF)、盐酸、甲醇、乙醇、甲苯、乙苯(分析纯,天津市科密欧化学试剂有限公司),苯(分析纯,天津市北联精细化学品开发有限公司), 5 μL GC微量进样器、不锈钢丝(上海高鸽工业贸易有限公司)。使用甲醇作为溶剂制备分析物的储备溶液并保存在冰箱中。使用前,通过用甲醇适当稀释其储备溶液来制备标准工作溶液。

### 1.2 样品采集

用聚氟乙烯Tedlar气体采集袋对呼出气样本进行采集。12份呼出气样本包括6名肺癌患者和6名健康志愿者。本研究经兰州大学附属第一医院临床研究与伦理委员会批准(编号:LDYYSZLL2022-02)。所有提供样本呼出气的志愿者在呼出气采集前至少禁食6 h,采集袋事先用氮气冲洗,以消除污染物。受试者用水清洁口腔,自由呼吸至少30 min后采样。收集的呼出气样品避光保存,以避免挥发性物质在分析前发生光氧化。进行SPME实验时,在抽真空的萃取瓶中加入5 mL的呼出气样品,每个样品重复测定3次。为了保证实验的准确性,所有样品需在24 h内完成检测。

### 1.3 仪器条件

色谱条件 TG-624 GC毛细管柱(60 m×0.25 mm×1.4 μm,美国赛默飞世尔科技公司);不分流模式,溶剂延迟时间为3 min;进样口温度260 ℃;载气为高纯氦气(99.999%,尚澜氦业,天津),流速为1.0 mL/min。

质谱条件 传输线和离子源温度均为250 ℃;选择性离子监测(SIM)模式;各分析物的监测离子*m/z*值如下:苯51、63、78,甲苯51、65、91,乙苯65、91、106;邻二甲苯77、91、106,癸醛57、70、82。

### 1.4 g-C_3_N_4_/UiO-66材料的合成

g-C_3_N_4_的制备:称取12 g尿素,平铺在石英舟中,将其转移到管式炉内,在空气氛围下加热至550 ℃,恒温4 h,然后冷却至室温,得到具有轻盈疏松结构的淡黄色固体g-C_3_N_4_。将其研磨后分散在甲醇溶液中超声处理2 h,使块状的g-C_3_N_4_充分剥离,将悬浮液6000 r/min离心10 min, 80 ℃烘干,得到g-C_3_N_4_备用。

g-C_3_N_4_/UiO-66的制备:将0.3500 g ZrCl_4_和0.2500 g对苯二甲酸溶解于75 mL的DMF溶液中,搅拌10 min,将溶液转移至容量为100 mL的聚四氟乙烯反应釜内,120 ℃反应48 h,放置至室温后离心分离。用DMF和乙醇分别洗涤3次,离心分离,120 ℃烘干。将UiO-66与g-C_3_N_4_以质量比10∶1在甲醇溶液中超声分散均匀,然后搅拌24 h, 100 ℃干燥得到g-C_3_N_4_/UiO-66复合材料。

### 1.5 固相微萃取纤维的制备

选用0.3 mm的不锈钢丝,用砂纸打磨任意一端,使其便于后续蚀刻步骤。在试管中配制约5 mL王水(HCl/HNO_3_=3∶1, v/v)。将不锈钢丝打磨的一端浸没在王水中5 min左右使其形成粗糙的表面,分别用乙醇和超纯水超声洗涤蚀刻部分3 min,自然风干。将不锈钢丝刻蚀的一端蘸取少量耐高温密封胶,用称量纸拭去多余胶体,使其在不锈钢丝表面形成胶膜,将其插入g-C_3_N_4_/UiO-66粉末中,通过旋转不锈钢丝均匀黏附涂层材料,自然风干8 h,待第一层材料定型固化后,重复操作两次,得到长度为2.5 cm、厚度为20~30 μm的纤维涂层。

### 1.6 固相微萃取实验

采用直接固相微萃取(DI-SPME)模式进行萃取实验。做标准曲线和加标试验时,用移液枪移取10 μL标准溶液注入25 mL的固相微萃取小瓶中;测定实际样品时,在抽真空的萃取瓶中加入 5 mL的呼出气样本。用自制固相微萃取装置的针头刺穿萃取小瓶的橡胶隔垫,然后推动针杆将涂层纤维于60 ℃暴露在气体基质中;纤维富集10 min后,将其迅速插入到气相进样口中于260 ℃解吸3 min,进行GC-MS分析检测。两次萃取实验之间应将固相微萃取纤维在进样口老化5 min,排除纤维在空气中吸附其他物质所造成的干扰。

## 2 结果与讨论

### 2.1 g-C_3_N_4_/UiO-66材料的表征

对g-C_3_N_4_、UiO-66以及复合材料g-C_3_N_4_/UiO-66进行了XRD衍射分析,结果如图1a所示,UiO-66具有明显的衍射峰,出峰位置与文献[29]一致;由于氮化碳的非晶体结构,衍射峰在27.4°处出现一个较宽的衍射峰,其他位置并没有明显的衍射峰出现。二者复合材料的峰形与UiO-66基本一致,由于g-C_3_N_4_非晶体结构的引入,衍射峰的强度与单一UiO-66相比略有降低,证明g-C_3_N_4_/UiO-66复合材料成功合成。

**图 1:**
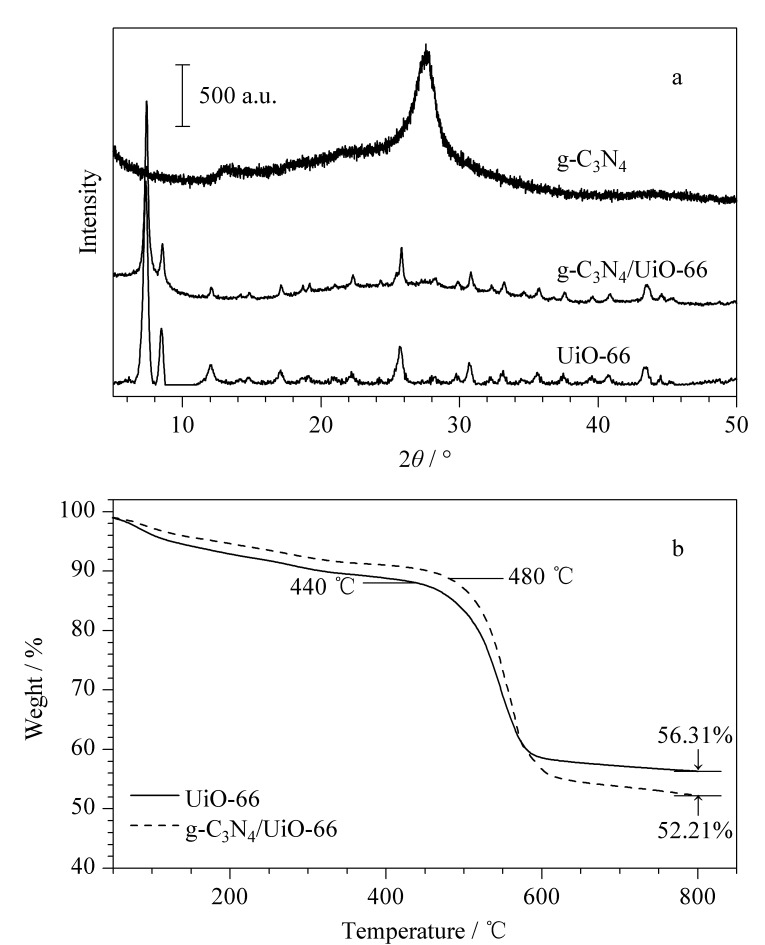
(a) UiO-66、g-C_3_N_4_和g-C_3_N_4_/UiO-66的XRD衍射谱;(b) UiO-66和g-C_3_N_4_/UiO-66的TGA曲线

g-C_3_N_4_的合成温度在550 ℃,当温度高于720 ℃才会发生热解。因此,g-C_3_N_4_具有优异的热稳定性,如图1b所示,与UiO-66复合之后材料在480 ℃之后才会有明显的质量损失,而单一的UiO-66在440 ℃时质量已损失10%。

材料的FT-IR表征如图2所示,复合材料在1000 cm^-1^和740 cm^-1^处出现UiO-66的特征吸收峰;在1250 cm^-1^和814 cm^-1^处与g-C_3_N_4_的红外特征峰一致,同样证明g-C_3_N_4_/UiO-66材料的成功合成。

**图 2:**
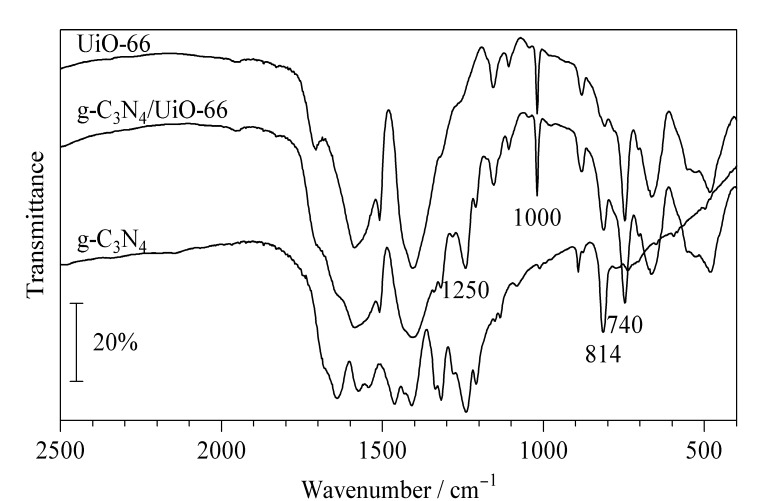
g-C_3_N_4_、UiO-66和g-C_3_N_4_/UiO-66的红外光谱图

尿素经过550 ℃的热处理,形成了由蓬松纳米片堆叠的纳米花状结构,如图3a所示,g-C_3_N_4_纳米片经过超声剥离变得更加分散,相互之间形成了丰富的层间通道。图3b为g-C_3_N_4_/UiO-66固相微萃取纤维的扫描电镜局部放大图像,UiO-66呈现出规则的正八面体结构,棱角分明,镶嵌在g-C_3_N_4_纳米花当中,表面被g-C_3_N_4_包裹,形成了疏水屏障。纤维的扫描电镜如图3c所示,涂层纤维均匀平整。

**图 3:**
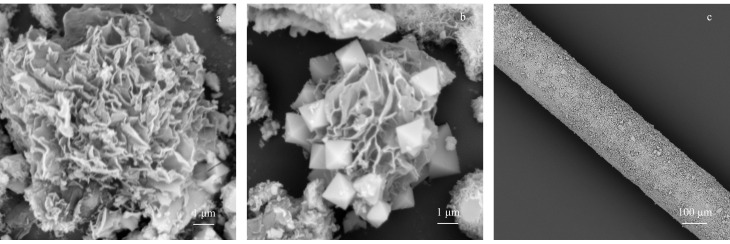
(a) g-C_3_N_4_、(b) g-C_3_N_4_/UiO-66和(c) g-C_3_N_4_/UiO-66固相微萃取纤维的扫描电镜图

### 2.2 萃取条件优化

为了保证g-C_3_N_4_/UiO-66固相微萃取纤维的萃取效率,以5种目标分析物的峰面积为响应值,使用标准溶液对萃取和解吸条件进行优化。如图4a所示,目标分析物的萃取峰面积在10 min基本趋于稳定,为了节约样品前处理时间,将最佳萃取时间确定为10 min。

**图 4:**
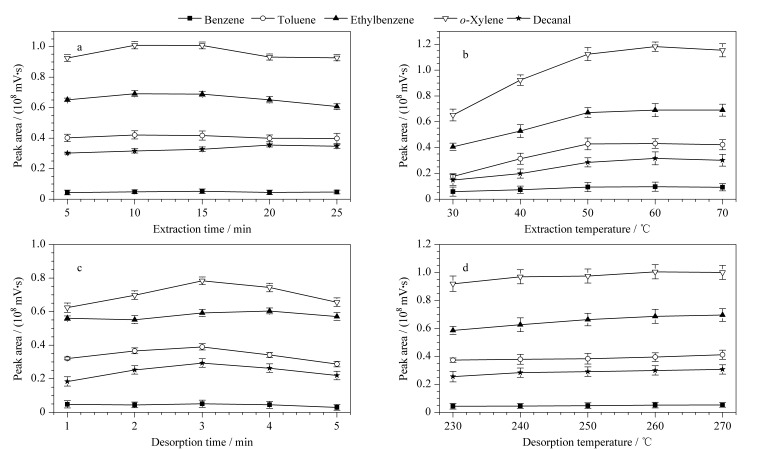
(a)萃取时间、(b)萃取温度、(c)解吸时间和(d)解吸温度对萃取效率的影响

萃取温度升高有利于低沸点微量液体样品迅速挥发为气态,并加速气态样品向纤维的扩散。如图4b所示,在30~50 ℃内随着萃取温度的升高,峰面积呈明显的上升趋势;60 ℃以后峰面积不再增加,因此,将60 ℃作为最佳萃取温度。

适当的解吸时间和解吸温度既可以使目标分析物完全解吸,又可以维持纤维的使用寿命。如图4c和4d所示,进样口解吸时间为3 min时效果最佳,而解吸温度设定在260和270 ℃时峰面积没有明显的变化。因此,将解吸时间和解吸温度分别确定为3 min和260 ℃。

### 2.3 萃取纤维的稳定性

每一次SPME过程萃取纤维都需要经过目标分析物的提取、GC进样口的高温解吸以及5 min的老化过程,过长时间的使用会造成纤维涂层不同程度的脱落。因此,纤维的稳定性对实验参数的测量、目标分析物的定量至关重要。为了考察多次重复萃取对纤维性能的影响,用同一根SPME纤维对目标分析物进行了120次萃取、解吸过程。结果如图5a所示,经过120次的连续萃取,纤维的萃取效率下降了7.62%~13.56%,萃取效率的相对标准偏差(RSD)为2.4%~6.5%,表明g-C_3_N_4_/UiO-66纤维涂层具有令人满意的循环稳定性。

**图 5:**
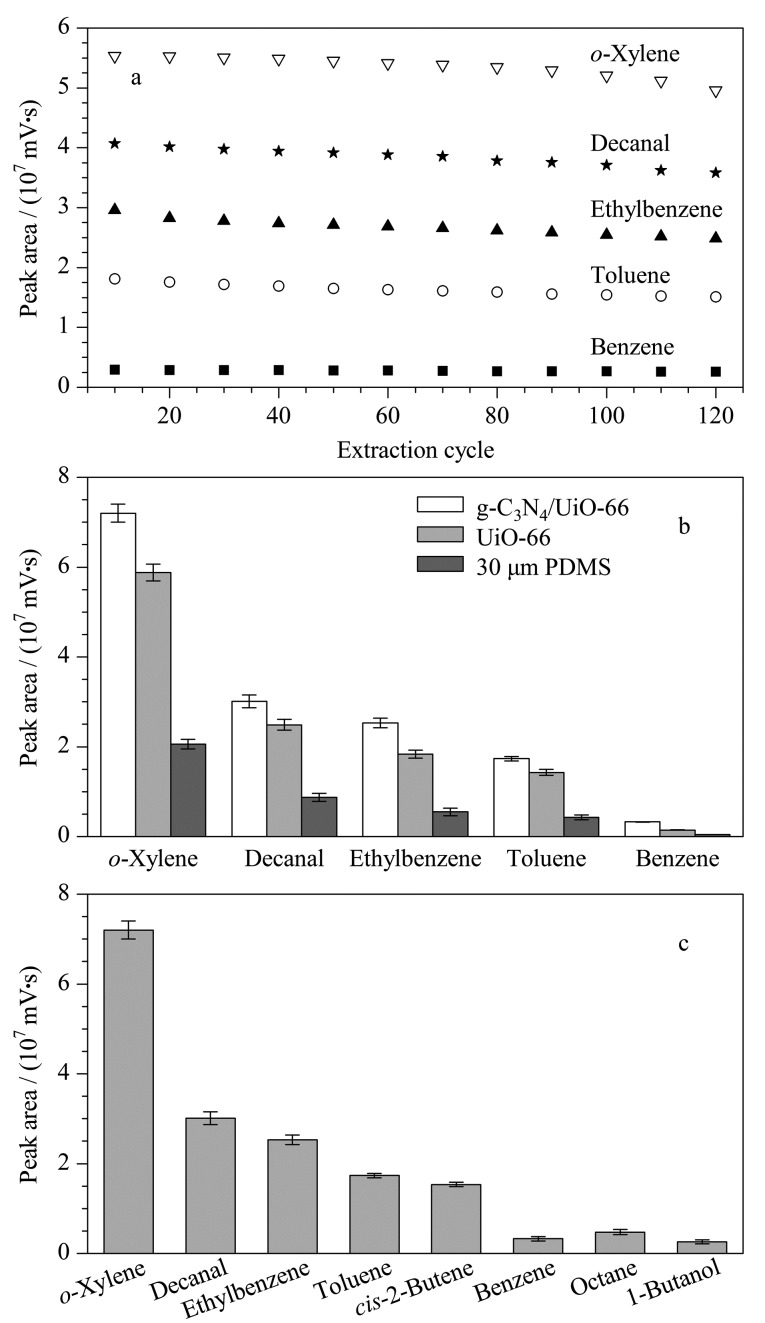
(a)涂层纤维对5种目标物的SPME稳定性,(b) g-C_3_N_4_/ UiO-66复合纤维与单一UiO-66纤维以及30 μm PDMS商用纤维比较(*n*=3),(c) g-C_3_N_4_/UiO-66复合纤维对不同生物标志物的吸附效果(*n*=3)

### 2.4 g-C_3_N_4_/UiO-66涂层纤维与商品化纤维的对比

将制备的g-C_3_N_4_/UiO-66纤维涂层与单一的UiO-66和30 μm PDMS商用纤维进行了比较。图5b显示了这3种纤维对5种目标分析物的萃取结果,g-C_3_N_4_的引入丰富了萃取纤维的比表面积,复合g-C_3_N_4_后涂层的萃取效率明显高于单一的UiO-66纤维涂层和PDMS商用纤维。商用PDMS的萃取峰面积仅为g-C_3_N_4_/UiO-66纤维涂层的1/4左右。

### 2.5 萃取机理探讨

如图3所示,g-C_3_N_4_具有纳米级的微孔,纳米片堆叠形成丰富的层间通道,与UiO-66复合之后,有利于加快目标分析物的传质过程,缩短SPME的萃取时间(仅需10 min达到萃取平衡)。正八面体MOF镶嵌在纳米花当中,为萃取纤维提供了丰富的比表面积(2236 m^2^/g)。如图5c所示,与其他不含苯环的肺癌标志物顺式-2-丁烯、正辛烷、1-丁醇相比,萃取纤维对甲苯、乙苯和邻二甲苯的萃取效率明显较好,这是由于g-C_3_N_4_氮元素的引入有利于与苯环类目标分析物形成*π-π*相互作用,可增强目标VOCs的富集效率。

### 2.6 方法学评价

#### 2.6.1 相关系数、检出限和定量限

使用GC-MS对苯、甲苯、乙苯、邻二甲苯和癸醛5种VOCs进行分析,结果表明,苯在10~2000 μg/L、甲苯在5~1000 μg/L、其他3种生物标志物在5~2000 μg/L范围内均呈良好的线性关系,相关系数(*r*^2^)为0.9919~0.9977(表1)。以空白样品加标测定5种VOCs,检出限(LOD, *S/N*=3)为0.16~4.50 μg/L,定量限(LOQ, *S/N*=10)为0.54~9.18 μg/L。

**表1:** 5种生物标志物的线性范围、相关系数、检出限、定量限和精密度

Analyte	Linear range/ (μg/L)	*r*^2^	LOD/ (μg/L, *S/N*=3)	LOQ/ (μg/L, *S/N*=10)	RSDs/%		
					Intra-day (*n*=6)	Inter-day (*n*=6)	Fiber-to-fiber (*n*=3)
Benzene	10-2000	0.9925	4.50	9.18	3.3	5.0	8.8
Toluene	5-1000	0.9941	0.83	2.79	5.6	4.4	10.1
Ethylbenzene	5-2000	0.9977	0.44	1.46	7.3	5.8	9.0
*o*-Xylene	5-2000	0.9919	0.16	0.54	5.8	4.0	7.8
Decanal	5-2000	0.9934	0.45	1.50	4.9	6.6	13.0

#### 2.6.2 精密度

在空白样品中加入5种VOCs的混合标准溶液进行测定,同一纤维6次平行测定的日内和日间精密度的RSD分别为3.3%~7.3%和4.0%~6.6%。3根平行制备的SPME纤维间的RSD为8.8%~13.0%。

### 2.7 实际样品分析及方法准确度

为了验证该方法的可行性,在最佳实验条件下对采集的呼出气样品进行了SPME分析。在健康志愿者的呼出气中未检测到邻二甲苯,无论是健康人群还是肺癌患者,呼出气中苯的含量普遍较低,而6名肺癌患者呼出气中甲苯含量均高于健康人群。肺癌患者5种VOCs的检出含量如下:苯7.5~8.9 μg/L,甲苯28.2~100.2 μg/L,乙苯56.4~148.8 μg/L,邻二甲苯12.5~60.5 μg/L,癸醛94.2~153 μg/L。对5种标志物进行高、中、低3个水平下的加标试验,平均回收率为84.6%~113.2%(见表2)。实验结果表明,所建立的SPME-GC-MS具有良好的准确度,适用于人体呼出气中生物标志物的分析检测。

**表2:** 实际样品中5种VOCs的含量及加标回收率(*n*=6)

Analyte	Healthy people					Patients			
	Content/ (μg/L)	Recoveries at three levels/%				Content/ (μg/L)	Recoveries at three levels/%		
		10 μg/L	50 μg/L	100 μg/L			10 μg/L	50 μg/L	100 μg/L
Benzene	5.6±0.6	92.3±4.2	95.8±6.6	89.4±5.3		8.2±0.7	96.3±8.0	103.1±4.6	90.5±6.4
Toluene	13.5±11.3	94.7±2.4	105.0±5.3	85.2±4.2		64.2±36.0	92.5±9.2	93.5±7.7	105.0±6.5
Ethylbenzene	80.4±32.0	85.3±3.8	94.1±8.0	104.2±6.1		102.6±46.2	102.1±6.0	84.6±6.1	87.4±4.6
*o*-Xylene	<LOD	93.8±3.0	99.3±7.0	113.2±8.9		36.5±24.0	88.0±7.3	102.2±5.8	91.2±7.5
Decanal	93.2±16.0	100.4±5.2	89.6±8.4	106.4±6.5		123.6±29.4	105.0±3.5	112.4±5.2	89.4±6.1

## 3 结论

本研究将g-C_3_N_4_与金属有机骨架UiO-66复合,改善了MOFs直接作为固相微萃取纤维的稳定性,联用GC-MS技术对肺癌患者呼出气中的5种VOCs进行了分析检测,并对萃取条件进行了系列优化。该方法具有较宽的线性范围、较低的检出限和定量限、较高的准确度,有望为肺癌患者的早期诊断提供更便捷的临床检测手段。

## References

[1] Danaher P J, Phillips M, Schmitt P, et al. Open Forum Infect Dis, 2022, 9(10): 489 10.1093/ofid/ofac489 PMC9578165 36267247

[2] Phillips M, Gleeson K, Hughes J M, et al. Lancet, 1999, 353(9168): 1930 10.1016/S0140-6736(98)07552-7 10371572

[3] Azim A, Barber C, Dennison P, et al. Eur Respir J, 2019, 54(3): 56 10.1183/13993003.00056-2019 31273044

[4] Schleich F, Zanella D, Dallinga J, et al. Am J Respir Crit Care Med, 2019, 200: 444 30973757 10.1164/rccm.201811-2210OC

[5] Wilson A D. Metabolites, 2015, 5(1): 140 10.3390/metabo5010140 PMC4381294 25738426

[6] Yodsin N, Sriphumrat K, Mano P, et al. Microporous Mesoporous Mater, 2022, 343: 112187 35999991 10.1016/j.micromeso.2022.112187 PMC9389852

[7] Ma H Y, Li X, Che J M, et al. Anal Methods, 2014, 6(17): 6841

[8] Atanu1 B, Nae-Eung L. Adv Mater Technol, 2021, 6(3): 1

[9] Chen T, Liu T N, Li T, et al. Clin Chim Acta, 2021, 515: 61 33387463 10.1016/j.cca.2020.12.036

[10] Arthur C L, Pawliszyn J. Anal Chem, 1990, 62(19): 2145

[11] Jia Y, Qian J, Pan B. Anal Chem, 2021, 93(32): 11116 10.1021/acs.analchem.1c01489 34346203

[12] Iranmanesh M, Ezzatpanah H, Akbari-Adergani B, et al. Int J Food Prop, 2018, 21(1): 1067

[13] Huang Y, Fang S, Xiang Z, et al. Sci Total Environ, 2022, 845: 335 10.1016/j.scitotenv.2022.157335 35842160

[14] Gallidabino M D, Bylenga K, Elliott S, et al. Anal Bioanal Chem, 2022, 414(17): 4987 10.1007/s00216-022-04129-w PMC9234032 35608670

[15] Cancelli A M, Gobas F. Environ Biosaf Res, 2022, 213: 755 10.1016/j.envres.2022.113755 35753377

[16] Ren J, Lu Y, Han Y, et al. Food Chem, 2023, 400: 4062 10.1016/j.foodchem.2022.134062 36075165

[17] Alimzhanova M, Mamedova M, Ashimuly K, et al. Food Chem-x, 2022, 14: 345 10.1016/j.fochx.2022.100345 PMC9156867 35663598

[18] Xu S, Liu H, Chen C, et al. Chem Eng J, 2023, 451: 569

[19] Hasani F, Raoof J B, Ghani M, et al. Mikrochim Acta, 2022, 189(11): 432 10.1007/s00604-022-05537-6 36284019

[20] Han H, Ding S, Geng Y, et al. Food Chem, 2023, 403: 134310 36156398 10.1016/j.foodchem.2022.134310

[21] Yang Z, Guo Z, Zhang J, et al. Res Chem Intermed, 2021, 47(1): 325

[22] Zhang J, Hu Y, Qin J, et al. Chem Eng J, 2020, 385: 123814

[23] Dong M, Wang X, Wu C, et al. Adv Funct Mater, 2020, 30 (7): 1908519

[24] Zhang H, Zhan G, Liu Z, et al. Chem-Asian J, 2021, 16 (11): 1499 10.1002/asia.202100262 33871155

[25] Raza W, Kukkar D, Saulat H, et al. TrAC-Trends Anal Chem, 2019, 120: 115654

[26] Zhang W, Hu Y, Ge J, et al. J Am Chem Soc, 2014, 136 (49): 16978 10.1021/ja509960n 25412280

[27] Chen K, Wu C. Angew Chem Int Ed, 2019, 58(24): 8119 10.1002/anie.201903367 30977951

[28] Reyes-Garces N, Gionfriddo E, Gomez-Rios G A, et al. Anal Chem, 2018, 90(1): 302 10.1021/acs.analchem.7b04502 29116756

[29] Shi L, Wang T, Zhang H, et al. Adv Funct Mater, 2015, 25(33): 5360

